# A bioactive heart-derived ECM hydrogel potentiates Astragaloside IV–mediated microvascular regeneration

**DOI:** 10.3389/fbioe.2026.1800990

**Published:** 2026-04-08

**Authors:** Yanxing Lan, Zijun Xu, Yajing Li, Dahe Liu, Wenguang Lu, Zhangwei Li, Peier Chen, Guoqin Chen

**Affiliations:** 1 Postgraduate Cultivation Base of Guangzhou University of Chinese Medicine, Panyu Central Hospital, Guangzhou, China; 2 The Tenth Affiliated Hospital (Dongguan People’s Hospital), Southern Medical University, Dongguan, China; 3 The Affiliated Dongguan Songshan Lake Center Hospital, Dongguan, China; 4 South China Normal University, Guangzhou, China; 5 Guangdong Provincial Key Laboratory of Cardiac Function and Microcirculation, Guangzhou, China; 6 Cardiovascular Diseases Research Institute of Panyu District, Guangzhou, China; 7 The Affiliated Panyu Central Hospital, Guangzhou Medical University, Guangzhou, China

**Keywords:** angiogenesis, Astragaloside IV, EGFR/Raf-MEK-ERK pathway, heart decellularized extracellular matrix, myocardial infarction

## Abstract

Impaired microcirculation and insufficient angiogenesis critically limit functional recovery after myocardial infarction (MI), yet effective strategies for localized and sustained vascular regeneration remain lacking. Here, we develop a heart-specific extracellular matrix–based hydrogel for the intrapericardial delivery of Astragaloside IV (AS-IV), a pro-angiogenic bioactive monomer derived from traditional Chinese medicine. The bioactive hydrogel is fabricated from decellularized porcine heart extracellular matrix (HdECM), which provides a biomimetic cardiac microenvironment while enabling sustained local release of AS-IV. Distinct from conventional intramyocardial injection, intrapericardial administration allows the hydrogel to form a patch-like depot on the cardiac surface, achieving prolonged cardiac retention with minimal tissue injury. Under oxygen–glucose deprivation, the AS-IV@HdECM hydrogel synergistically enhances endothelial cell survival, migration, and angiogenic capacity while suppressing apoptosis. In a rat MI model, localized delivery of AS-IV via HdECM markedly promotes neovascularization, attenuates myocardial fibrosis, reduces cardiomyocyte apoptosis, and significantly improves cardiac function. Mechanistically, integrative proteomic and network pharmacology analyses, together with experimental validation, indicate that the therapeutic effects are associated with activation of the EGFR/Raf–MEK–ERK signaling pathway. This work establishes a localized therapeutic paradigm that integrates heart-specific matrix cues with traditional Chinese medicine, offering a translational biomaterials strategy for microvascular regeneration and cardiac repair after MI.

## 1 Introduction

Myocardial infarction (MI) is characterized by profound microvascular injury and insufficient angiogenesis, which critically limit long-term functional recovery despite substantial advances in reperfusion therapies and pharmacological management (Virani et al., 2021; Grune et al., 2022; Prabhu and Frangogiannis, 2016). Although timely restoration of epicardial blood flow can alleviate acute ischemic damage, persistent impairment of the microcirculation compromises oxygen and nutrient delivery, exacerbates cardiomyocyte loss, and accelerates adverse ventricular remodeling, ultimately leading to heart failure (Cabac-Pogorevici et al., 2020; Wu et al., 2018). As functional revascularization is a critical prerequisite for effective myocardial repair and survival of cardiomyocytes, restoration of microvascular networks has emerged as a central objective in post-infarction cardiac repair.

During the early phase following MI, residual microvessels and endothelial cells retain partial regenerative capacity, providing a therapeutic window for microvascular regeneration through angiogenic intervention (Wu et al., 2021). However, existing approaches aimed at promoting neovascularization face substantial challenges. Systemic administration of pro-angiogenic agents typically suffers from poor cardiac targeting, rapid clearance, and off-target effects, resulting in limited therapeutic efficacy. In contrast, intramyocardial injection enables localized delivery but introduces mechanical injury, heterogeneous drug distribution, and inflammatory responses that may further compromise myocardial tissue (Li et al., 2022). These limitations highlight the absence of clinically translatable strategies for localized, sustained, and minimally invasive promotion of microvascular regeneration in the infarcted heart.

Traditional Chinese medicine (TCM) provides a rich source of bioactive compounds with multi-target therapeutic potential for cardiovascular diseases. Astragaloside IV (AS-IV) (Supplementary Figure S1), a major saponin extracted from Astragalus membranaceus, has been reported to primarily promote angiogenesis while exerting additional anti-inflammatory and cytoprotective effects (Xu et al., 2023; Sun et al., 2021; Zhang W. et al., 2022). At the molecular level, AS-IV modulates multiple signaling pathways associated with endothelial function, oxidative stress, and cell survival (Sui et al., 2019; Zhang X. et al., 2022; Yin et al., 2019). Despite these promising biological activities, the clinical translation of AS-IV remains limited by its poor water solubility, low bioavailability, and lack of tissue specificity (Zhang et al., 2006; Chang et al., 2012), which collectively restrict its therapeutic efficacy *in vivo*.

Recent advances in biomaterials-based drug delivery systems offer new opportunities to address these challenges. Bioactive hydrogels derived from decellularized extracellular matrix (dECM) have attracted increasing attention owing to their intrinsic bioactivity, tissue-specific composition, and excellent biocompatibility (Gil-Cabrerizo et al., 2023). In particular, heart-derived decellularized extracellular matrix (HdECM) preserves native cardiac biochemical cues that support endothelial survival, angiogenesis, and tissue remodeling (Wang B. et al., 2023; Song et al., 2023). Moreover, HdECM hydrogels undergo *in situ* gelation and can function as sustained-release depots for therapeutic agents (Gómez-Cid et al., 2021). Importantly, accumulating preclinical and clinical evidence has demonstrated the safety and feasibility of HdECM-based materials for cardiac applications (Traverse et al., 2019). Nevertheless, the use of HdECM hydrogels as delivery platforms for traditional Chinese medicine–derived small molecules in myocardial repair remains largely unexplored.

Here, we present a heart-specific extracellular matrix–based hydrogel system for the localized delivery of AS-IV to promote microvascular regeneration after MI. By encapsulating AS-IV within an bioactive HdECM hydrogel, this strategy enhances drug solubility, prolongs cardiac retention, and provides a biomimetic microenvironment conducive to angiogenesis. Importantly, intrapericardial administration represents a minimally invasive alternative to intramyocardial injection, enabling the hydrogel to form a patch-like depot on the cardiac surface with sustained local drug exposure.

**SCHEME 1:**
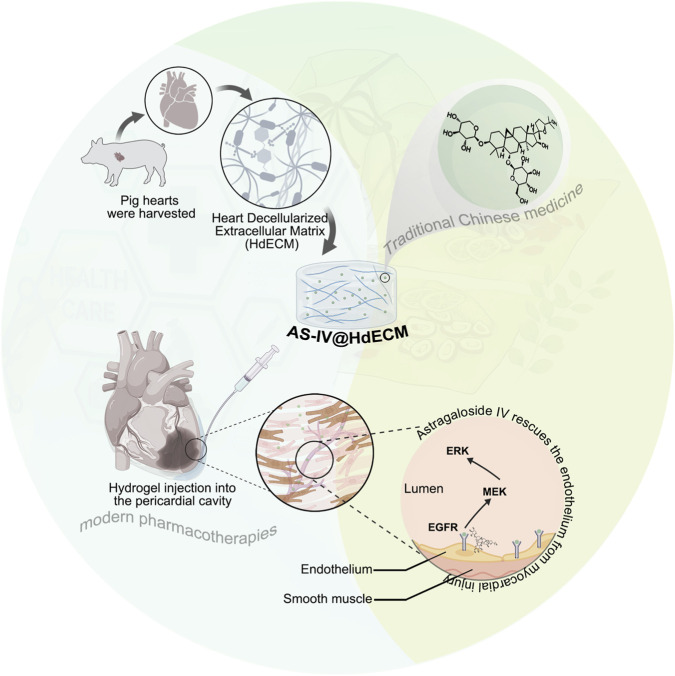
Schematic illustration of the AS-IV@HdECM for cardiac repair.

Through a combination of *in vitro* and *in vivo* studies, we demonstrate that AS-IV@HdECM synergistically enhances endothelial cell survival and angiogenesis, attenuates myocardial fibrosis and apoptosis, and significantly improves cardiac function after MI. Furthermore, integrative proteomic and network pharmacology analyses, together with experimental validation, indicate that activation of the EGFR/Raf–MEK–ERK signaling pathway is associated with the observed therapeutic effects. This work establishes a localized therapeutic paradigm that integrates heart-specific matrix cues with traditional Chinese medicine, offering a translational biomaterials-based strategy for microvascular regeneration and cardiac repair (Scheme 1).

## 2 Materials and methods

### 2.1 Materials

Astragaloside IV (purity ≥98%, source: Leaf) was purchased from Guangzhou Yuntian Laboratory Technology Co., Ltd. (China). Pepsin (enzyme activity: ≥2500 U/mg) was obtained from Shanghai Aladdin Biochemical Technology Co., Ltd. (China). Human umbilical vein endothelial cells (HUVECs) were purchased from Wuhan (China). EDU cell proliferation kit, and TUNEL cell apoptosis detection kit were purchased from Beyotime Biotechnology (China). Matrigel was purchased from Corning Inc. (USA). Masson’s trichrome staining kit and hematoxylin-eosin (H&E) staining kit were purchased from Shanghai Baiyuan Biotechnology Co., Ltd. (China). Enzyme-linked immunosorbent assay (ELISA) kits for rat creatine kinase-MB (CK-MB), cardiac troponin I (cTnI), interleukin-1β (IL-1β), interleukin-6 (IL-6), tumor necrosis factor-α (TNF-α), alanine aminotransferase (ALT), aspartate aminotransferase (AST), serum creatinine (Scr), and uric acid (UA) were purchased from Shanghai Enzyme-Linked Biotechnology Co., Ltd. (China). CD31, α-SMA, and Ki67+ antibodies were obtained from Abcam (USA). MEK, ERK, and GAPDH antibodies were purchased from Proteintech Group, Inc. (China).

### 2.2 Heart decellularized matrix hydrogel (HdECM)

#### 2.2.1 Preparation and characterization of HdECM hydrogel

Fresh hearts were harvested from Yorkshire farm pigs (body weight: 35–45 kg) and the left ventricular (LV) was isolated, then was cut into small blocks and immersed in a 1 wt% sodium dodecyl sulfate (SDS) solution in PBS for decellularization. After the complete decellarization, the HdECM was rinsed to remove SDS, freeze-dried and grinded into a fine powder. In order to liquify the HdECM, the HdECM powder was partially digested by pepsin. To do that, a 16 mg/mL HdECM solution was prepared and digested in a 1 mg/mL pepsin solution in 0.01 M hydrochloric acid (HCl) for 48 h at room temperature. The digestion was stopped by neutralizing the solution to pH7.4 with 1 M sodium hydroxide (NaOH), followed by diluting with 1×PBS to achieve the desired HdECM concentrations. For the preparation of HdECM hydrogel, HdECM in a scintillation vial was incubated at 37 °C for 1 h until the solution was resistant to flow upon tilting the vial. To load AS-IV into the HdECM hydrogel, a HdECM hydrogel was immersed in a solution containing certain amount of AS-IV. The AS-IV@HdECM hydrogel was successfully obtained after the solution was gently stirred at 37 °C for sufficient time to achieve the adsorption equilibrium.

#### 2.2.2 Characterization of extracellular matrix hydrogel

The morphological structure of HdECM and AS-IV@HdECM hydrogels was analyzed using Scanning Electron Microscope (SEM) after freeze-drying the samples. The swelling experiment was done by immersing dried hydrogel samples in PBS at 37 °C for at room temperature and the change in weight was measured until a stable weigh was reached. The storage modulus (G′) and loss modulus (G″) of the hydrogel were measured using a modular rheometer (Anton Paar MCR102e). The changes in G′ and G″ of each hydrogel group under different shear stresses were recorded. To evaluate the *in vitro* degradation rate of the hydrogel, the hydrogel was immersed in PBS at 37 °C. The remaining mass was weighed at regular intervals and fresh PBS was replaced after each weighing until the hydrogel was completely degraded.

#### 2.2.3 *In vitro* releasing profile of AS-IV

The AS-IV@HdECM hydrogel was placed into a dialysis bag and immersed in PBS at 37 °C. At predetermined time intervals, 3 mL of release medium was collected for analysis, and an equal volume of fresh PBS was added to maintain a constant volume. The content of AS-IV in the collected samples was quantified using high performance liquid chromatography (HPLC; Thermo Fisher Vanquish, USA) at an absorbance wavelength of 203 nm. The concentration of AS-IV was determined by comparing the sample data to a pre-established standard curve.

#### 2.2.4 Encapsulation efficiency and drug loading capacity

To assess the drug loading capacity, methanol was employed to extract AS-IV from a AS-IV@HdECM hydrogel of known weight. Then, the amount of AS-IV in the methanol extract was determined using HPLC, based on a pre-established standard curve.

#### 2.2.5 Cytotoxicity assessment of HdECM

H9c2 cells were used here to assess the cytotoxicity of HdECM. HdECM hydrogel solutions were prepared at a range of concentrations, specifically 0, 2, 4, 8, 12,16 and 20 mg/mL to evaluate their effect on H9c2 viability and CCK-8 assay was performed to assess the cell viability.

### 2.3 *In vitro* cell experiment

#### 2.3.1 Cell viability tests against AS-IV

The effect of AS-IV as well as HdECM on the cell viability of HUVEC cells was investigated separately. The concentrations studied for HdECM was specifically 0, 2, 4, 6, 8,10 and 12 mg/mL and that for AS-IV was 0, 20, 40, 80, 120, 160, 200 μM. HUVEC cells were exposed to these varying concentrations of HdECM hydrogel or AS-IV for 24 h and the cell viability was assessed using a CCK-8 assay kit.

#### 2.3.2 Establish the oxygen-glucose deprivation (OGD) cell model

The OGD cell model was created by culturing HUVEC cells under hypoxic conditions (94% N2, 5% CO2, 1% O2, 37 °C) using low-glucose DMEM medium without FBS. Following this, HUVECs were treated with various concentrations of AS-IV (low: 20; medium: 80; high: 160 μM) and AS-IV@HdECM (optimal concentration of HdECM) for 8 h under the same OGD conditions. The optimal concentrations of AS-IV and AS-IV@HdECM based on cell viability were selected for subsequent experiments. HUVECs were seeded in 24-well plates and allowed to adhere for 24 h. The optimal concentrations of HdECM, AS-IV, and AS-IV@HdECM were prepared using low-glucose DMEM without FBS. Different drug treatments were added to the respective wells, while the control group received only low-glucose DMEM without FBS. The cells were then transferred to a hypoxic cell culture incubator for 6–8 h of incubation. In the normal group, the medium was replaced with high-glucose DMEM containing 10% FBS, and the cells were cultured under normoxic conditions.

#### 2.3.3 Cell proliferation, migration and tube formation assays

HUVECs were divided into five groups: the normal control group, the OGD control group, the HdECM hydrogel group, the AS-IV group, and the AS-IV@HdECM hydrogel group. Following drug treatment under OGD conditions, EDU-labeled cells were incubated for an additional 2 h in a hypoxic incubator. After fixation and staining, the cells were imaged using a fluorescence microscope, and the number of proliferating cells was quantified.

When HUVECs reached over 100% confluence, a 200 µL pipette tip was used to create two to three parallel scratches perpendicular to the cell monolayer in each well. The cells were then treated with the respective drugs and transferred to a hypoxic incubator for further incubation. Scratch width was measured at 0, 12, 24, and 36 h using an inverted microscope.

Matrigel was thawed overnight at 4 °C and evenly coated onto the bottom of 96-well plates. HUVECs from different treatment groups were seeded onto the Matrigel-coated plates, which were then transferred to a hypoxic incubator and cultured for 8 h. Images were captured under a microscope and the results were recorded.

#### 2.3.4 TUNEL staining

HUVECs were uniformly seeded into 24-well plates and treated with the respective drugs for 8 h under OGD conditions. Subsequently, the cells were fixed with 4% PFA for 30 min and permeabilized with 0.3% Triton X-100 for 5 min at room temperature. After washing with PBS, the cells were incubated with TUNEL detection solution in the dark at 37 °C for 1 h. After DAPI staining, the cells were imaged using a fluorescence microscope, and the number of TUNEL-positive cells was quantified.

### 2.4 Animal experiment

#### 2.4.1 Myocardial infarction model construction

All animal experiments were approved by the Ethics Committee of Dongguan People’s Hospital (Approval Number: IACUC-AWEC-202204001). Male Sprague Dawley (SD) rats, aged 6–8 weeks and weighing 220 ± 20 g. The rats were anesthetized with 2% isoflurane using an anesthesia machine, intubated and connected to a ventilator. In the Sham group, a thoracotomy was performed without ligation of the left anterior descending (LAD) coronary artery. In the model group, the LAD coronary artery was permanently ligated with a 6–0 polypropylene suture. Following LAD ligation, rats were randomly assigned to receive 100 μL of PBS (MI group), HdECM hydrogel, AS-IV, or AS-IV@HdECM hydrogel via an injection into the pericardial cavity. Once the rats resumed spontaneous breathing and normal activity, they were returned to their cages and provided with a standard diet.

#### 2.4.2 Histological experiments and analysis

On day 28 post-myocardial infarction, heart tissues were immunostained for CD31 and α-SMA to evaluate angiogenesis. The mean fluorescence intensity in terms of quantitative data. Cardiomyocytes apoptosis at the infarct site was assessed using TUNEL staining. Masson’s trichrome and hematoxylin and eosin (H&E) staining were performed to evaluate the degree of fibrosis and cardiac morphology, respectively. Cell proliferation at the infarct border zone was assessed by immunohistochemical staining for Ki67+. Additionally, major organs, including the liver, spleen, lungs, and kidneys, were harvested and subjected to H&E staining to evaluate the biosafety profile of the compounds.

#### 2.4.3 *In vivo* imaging *in vitro*

HdECM hydrogel was labeled with rhodamine B and intrapericardial injections were administered to the rats in the respective treatment hydrogels. On days 1, 3, and 7 post-injection, the retention of the compounds in the pericardial cavity was monitored using a small animal *in vivo* fluorescence imaging system (PerkinElmer IVIS Spectrum). Fluorescence signals were quantified and analyzed using Living Image software.

#### 2.4.4 Measurement of cytokines in serum by ELISA

On the second day post-operation, blood samples were collected from the orbital vein to measure serum levels of CK-MB, cTnI, TNF-α, IL-6, and IL-1β.The concentrations factors including there biomarker in rat serum were quantified using a commercially available ELISA kit. On day 28 post-myocardial infarction, serum samples were collected for biochemical analysis, including measurements of ALT, AST, UA, and Scr.

#### 2.4.5 Echocardiography and hemodynamic assessment

The Vevo 3100LT system (Fujifilm VisualSonics, Inc.) was utilized M-mode and B mode echocardiography data were recorded to assess left ventricular function on days 14 and 28 post-MI. Rats were anesthetized with 2% isoflurane. Cardiac systolic and diastolic functions were evaluated by measuring key parameters, including left ventricular ejection fraction (LVEF), left ventricular fractional shortening (LVFS), left ventricular internal diameter at end-systole (LVIDs), and left ventricular internal diameter at end-diastole (LVIDd).

#### 2.4.6 Western blot

After drug treatment, myocardial tissues were harvested from the infarct border zone. Proteins were extracted using RIPA buffer supplemented with a protease inhibitor cocktail, and their concentrations were quantified using BCA protein assay kit. Following standard Western blot protocols, equal amounts of protein samples (40 μg) were electrophoresed on 10% SDS-polyacrylamide gels. The proteins were then transferred onto polyvinylidene difluoride (PVDF) membranes using a transfer buffer. The PVDF membranes were then incubated with primary antibodies at 4 °C overnight (12–16 h), followed by incubation with horseradish peroxidase (HRP)-conjugated secondary antibodies for 2 h at room temperature. The primary antibodies used included MER (1:5000), ERK (1:1000), and GAPDH (1:2000). Protein bands were visualized using a chemiluminescent HRP substrate and imaged using the VisionWorks system.

#### 2.4.7 q-PCR validation

Total RNA was extracted using TRIzol reagent (Invitrogen,USA) and reverse-transcribed into cDNA using a reverse transcription kit. The mRNA expression levels of target genes, including MER, ERK, Raf, and Elk-1, were quantified by quantitative qPCR and normalized to the housekeeping gene GAPDH. The primer sequences were as follows: Supplementary Table S1.

### 2.5 Network pharmacology analysis

#### 2.5.1 Proteomics analysis of HdECM hydrogel

Mass spectrometry analysis was conducted by Qingdao Standard Testing Co., Ltd. For protein identification, the enzymatic hydrolysate was separated using nano-scale liquid chromatography and subsequently analyzed with a Thermo QE HF mass spectrometer (Thermo Fisher). For data analysis, the raw mass spectrometry data were processed and searched against the relevant database using Proteome Discoverer 2.5 software (Thermo Fisher), yielding the protein identification data.

#### 2.5.2 Predict potential targets

The chemical and pharmacological information of AS-IV was retrieved from the TCMSP (https://old.tcmsp-e.com/tcmsp.php) and PubChem (https://pubchem.ncbi.nlm.nih.gov/) databases. AS-IV, along with the previously identified HdECM protein components, was submitted to the PharmMapper (http://lilab-ecust.cn/pharmmapper/index.html) and SwissTargetPrediction (http://swisstargetprediction.ch/) databases to predict potential molecular targets of AS-IV. Genes associated with MI were retrieved from the GeneCards (https://www.genecards.org/) database. The predicted targets of HdECM and AS-IV were cross-referenced with MI treatment targets using a Venn diagram (Venny 2.1.0) to identify shared therapeutic targets. The intersection genes were subsequently imported into the STRING (https://string-db.org/) database to construct a protein-protein interaction (PPI) network of HdECM, AS-IV, and AS-IV@HdECM. Following this, the data were imported into Cytoscape 3.9.0 software to identify core genes based on degree values and visualize the PPI network of key targets involved in the treatment of MI by these compounds.

#### 2.5.3 Network analysis

The core genes were analyzed using the DAVID (https://david.ncifcrf.gov/home.jsp) database, with the species set to *Rattus norvegicus*, to perform enrichment analysis of Gene Ontology (GO) biological processes (BP), cellular components (CC), molecular functions (MF), and KEGG signaling pathways. The top 20 GO terms and the highest-ranked KEGG pathways were visualized using Microbiomics and Cytoscape 3.9.0 software, revealing key signaling pathways associated with the therapeutic effects of these compounds in the treatment of MI.

#### 2.5.4 Molecular docking validation

First, the three-dimensional structures of the core target genes were retrieved from the PDB database (http://www.rcsb.org/) and used as protein receptors. Next, AS-IV, identified from the PubChem database (https://pubchem.ncbi.nlm.nih.gov/), was used as the ligand, and its three-dimensional structure was generated using Chem3D software. Subsequently, molecular docking was performed using AutoDock Vina to calculate the binding affinity between the protein receptors and the ligand (AS-IV). Finally, the docking results were visualized and analyzed using PyMOL software to assess the interaction between the protein and AS-IV.

### 2.6 Statistical analysis

Data analysis was performed using GraphPad Prism 9.3 (Inc., USA). Normality tests were first conducted to assess the distribution of the data. For comparisons between two groups, t-test was applied for normally distributed data, while the Mann-Whitney U test was used for non-normally distributed data. For comparisons involving three or more groups, one-way analysis of variance (ANOVA) was used for normally distributed data. For non-normally distributed data, the Kruskal–Wallis test was applied. Statistical significance was defined as a P-value <0.05. All data are expressed as mean ± standard deviation (SD).

## 3 Results and discussion

### 3.1 Preparation and characterization of hydrogel

**FIGURE 1:**
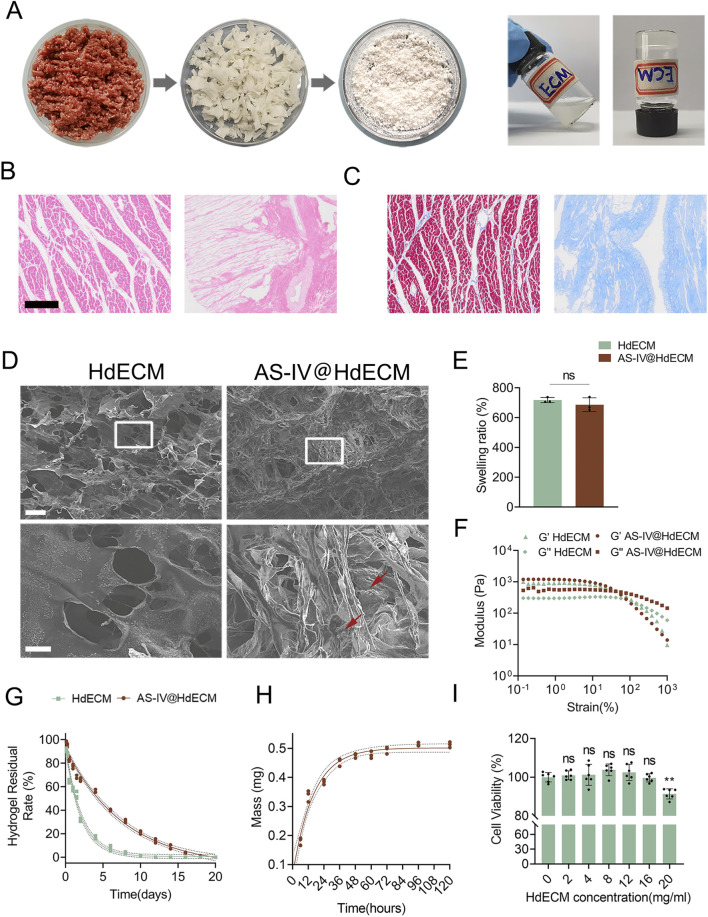
Preparation and characterization of HdECM and AS-IV@HdECM hydrogels. **(A)** Schematic illustration of the preparation process of HdECM hydrogel. **(B)** H&E and **(C)** Masson’s trichrome staining of porcine cardiac tissue before and after decellularization. (Scale bar:200 μm) **(D)** SEM cross-sectional images of HdECM and AS-IV@HdECM hydrogels. (Scale bars: 200μm and 50 μm) **(E)** Swelling rates of HdECM and AS-IV@HdECM hydrogels (n = 3). **(F)** Rheological analysis of HdECM and AS-IV@HdECM hydrogels. **(G)** Degradation rate of AS-IV@HdECM hydrogel (n = 3). **(H)** The release profile of AS-IV from AS-IV@HdECM hydrogel (n = 3). **(I)** Cytocompatibility of HdECM hydrogel (n = 6). “ns” indicates not significant. The symbol * indicates a significant difference compared to 0 mg/mL. Data were considered statistically significant at *p < 0.05, **P < 0.01, and ***P < 0.001 versus the indicated group.

The HdECM hydrogel used here was derived from decellularized porcine myocardium, following established methods from the literature (Ungerleider et al., 2015; Spang et al., 2022). Its formation occurs through a self-assembly process upon raising the temperature to 37 °C, making it an bioactive hydrogel (Figure 1A). It indicates that the hydrogel undergoes a solution-gel transformation due to temperature changes and can stably adhere to objects. H&E and Masson’s trichrome staining confirmed that the cells were almost completely removed (Figures 1B,C). SEM images of the lyophilized HdECM hydrogel’s cross-sectional area revealed a typical porous structure with uniform pore sizes ranging about 56.3 ± 19.4 μm. Upon drug loading with AS-IV, the porous architecture was retained in the AS-IV@HdECM hydrogel, though the structure became more compact, showing reduced pore size ranging about 39.1 ± 11.7μm, and a more regular pore arrangement (Figure 1D). This change in structure corresponds the swelling test results, where the swelling rate of HdECM hydrogel reached approximately 800%, while the AS-IV@HdECM hydrogel exhibited a lower swelling rate (Figure 1E). The reduced swelling rate can be attributed to the denser structure formed due to the interaction between the hydrophobic AS-IV and the HdECM matrix, which also contributes to and increase in the hydrogel’s mechanical strength.

An important feature of HdECM hydrogels is their ability to mimic the viscoelastic properties of biological tissues. To evaluate the viscoelastic properties of both HdECM and AS-IV@HdECM hydrogels, rheological measurements were performed under amplitude sweep conditions. The storage modulus (G′) of the HdECM hydrogel was just below 10^3^ Pa, with gel-sol transformation occurs at a strain rate of approximately 150%. In contrast, the AS-IV@HdECM hydrogel showed a slightly higher G' (>10^3^ Pa), indicating a stiffer material due to the mutual interactions between AS-IV and the hydrogel matrix. The loss modulus (G″) of AS-IV@HdECM hydrogel also increased upon AS-IV loading, leading to a decrease in the strain rate for gel-sol transformation to 41.8% (Figure 1F). These results both hydrogels possess suitable mechanical properties to function as bioactive drug delivery systems for MI treatment, especially considering the shear stress conditions of the pericardial cavity (Jacot et al., 2010; Chaudhuri et al., 2020).

Regarding biodegradation, HdECM underwent slow degradation in PBS, mainly due to hydrolysis and the potential activity of matrix metalloproteinases. Complete degradation occurred in approximately 8 days, consistent with previous studies (Wang et al., 2022). However, the degradation of AS-IV@HdECM was significantly slower, lasting around 16 days, likely due to the more compact porous structure and the presence of AS-IV (Figure 1G). This extended degradation rate is beneficial for long-term drug release when the AS-IV@HdECM hydrogel is injected into the body, although degradation could be accelerated *in vivo* such as enzymes (Zhang, 2021).

The release of AS-IV from AS-IV@HdECM hydrogel in PBS was tested, showing a slow and sustained release over 120 h of the loaded AS-IV being released during this period (Figure 1H). At an encapsulation efficiency of 72%, the release profile supports the potential of this hydrogel as an effective drug delivery system.

Finally, the cytocompatibility of HdECM hydrogel was assessed by exposing H9c2 cells to varying concentrations of the hydrogel. No significant toxicity was observed up to 20 mg/mL, a concentration much higher than that used in the bioactive hydrogels (Figure 1I). Thus, these results demonstrate the biocompatibility of the hydrogel and its suitability for biomedical applications.

These results demonstrate that the HdECM hydrogel can encapsulate, disperse, and solubilize AS-IV through its three-dimensional porous network, proteins and polysaccharides components, and high water content, thereby inhibiting drug aggregation and crystallization and improving its apparent solubility and stability. The thermosensitive hydrogel forms a denser network structure with suitable mechanical properties for *in situ* pericardial injection, enabling it to resist shear stress within the pericardial cavity. Furthermore, its excellent drug release profile and favorable biocompatibility further validate its potential as a bioactive and effective sustained-release delivery system for myocardial infarction therapy.

### 3.2 AS-IV@HdECM hydrogel induces HUVEC proliferation, migration, tube formation, and reduces apoptosis in an *in vitro* OGD model

#### 3.2.1 Screening for optimal drug concentrations

**FIGURE 2:**
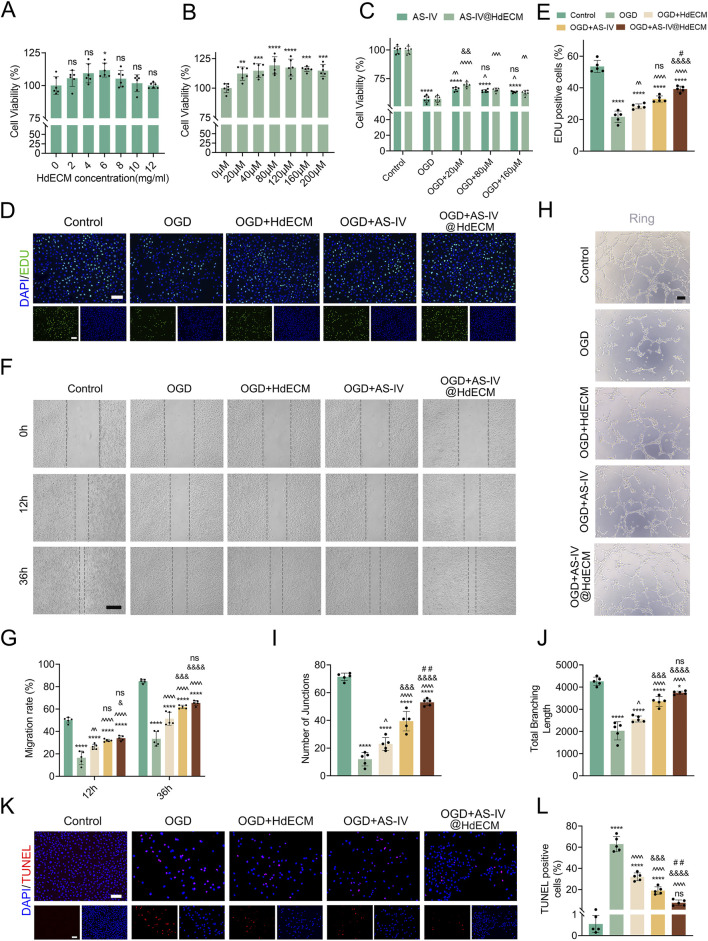
Effects of AS-IV@HdECM hydrogel on HUVECs *in vitro*. **(A,B)** Viability of HUVECs treated with different concentrations of HdECM hydrogel (n = 6) and with different concentrations of AS-IV (n = 6). **(C)** Viability of HUVECs treated with AS-IV and AS-IV@HdECM hydrogel under OGD conditions (n = 6). **(D)** EDU staining (scale bar: 250 μm) and **(E)** quantitative analysis of HUVECs proliferation (n = 5). **(F)** Migration images of HUVECs at 0, 12, and 36 h (scale bar:100 μm) and **(G)** statistical analysis (n = 5). **(H)** Images of HUVECs tube formation (scale bar:100 μm). **(I)** Quantitative analysis of number of nodes and **(J)** total branch length in HUVEC tube formation (n = 5). **(K)** TUNEL staining images showing changes in cell apoptosis (scale bar:100 μm) and **(L)** quantitative analysis of TUNEL-positive cells (n = 5). All bar graphs display individual data points. Symbols *, ^, &, and # denote comparisons with the Control group, OGD group, HdECM hydrogel group, and AS-IV group, respectively. “ns” indicates no significance; *p < 0.05, **P < 0.01, ***P < 0.001, ****P < 0.0001.

Previous studies have shown that AS-IV can enhance the activity of various cell types at certain concentrations (Cheng et al., 2019). In this study, we investigated the effect of AS-IV on HUVEC cells and observed that AS-IV increased HUVEC cell activity at all concentrations tested (Figures 2A,B). Additionally, HdECM hydrogel alone demonstrated a stimulatory effect on HUVEC proliferation, with the strongest effect observed at a concentration of 6 mg/mL. For the AS-IV@HdECM hydrogel, we fixed the HdECM concentration at 6 mg/mL and found that HUVEC cell activity peaked after 24 h of incubation when the AS-IV loading was 20 μM. This concentration produced the highest cell activity compared to medium (80 μM) and high (160 μM) AS-IV concentrations (Figure 2C). Based on these findings, the composition of 6 mg/mL HdECM and 20 μM AS-IV was selected for subsequent experiments.

The cellular microenvironment is marked by acute ischemia and hypoxia, which result from interrupted blood supply in MI. These conditions lead to reduced cell activity and, in severe cases, cell death. To explore whether AS-IV@HdECM hydrogel could mitigate such damage, we established an OGD model using HUVECs, simulating the ischemic conditions of MI. Cell activity was assessed by EDU staining, which labels newly synthesized DNA and identifies proliferating cells (Figure 2D). The proliferation rate of HUVECs was significantly lower in the OGD model compared to the control group, as evidenced by fewer EDU-positive cells (Figure 2E). However, treatment with the optimal AS-IV@HdECM hydrogel formulation notably increased the number of EDU-positive cells. Interestingly, both AS-IV and HdECM alone also promoted HUVEC proliferation under OGD conditions to some extent. These results suggests a potential synergistic effect between AS-IV and HdECM hydrogel, which together enhance cell proliferation more effectively than either component alone.

#### 3.2.2 Effects on endothelial cell migration, *in vitro* angiogenesis, and apoptosis

Endothelial cell migration, tube formation, and angiogenesis are interrelated processes essential for tissue repair. Newly formed blood vessels deliver oxygen and nutrients to ischemic regions, alleviating cellular damage (Wu et al., 2021; Bianconi et al., 2018). To assess cell migration, scratch assays were performed on a HUVEC monolayer under OGD conditions (Figure 2F). The wound closure rates were 51.4% for the HdECM hydrogel group, 61.9% for the AS-IV group, and 65.4% for the AS-IV@HdECM hydrogel group. All treatment groups exhibited a significant increase in migration compared to the OGD model group 33.4% (Figure 2G). While AS-IV’s ability to promote cell migration has been reported previously,our results show that this effect is enhanced when AS-IV is combined with HdECM hydrogel, which provides a favorable environment for endothelial cell proliferation and migration.

To further evaluate the angiogenic potential, we examined the tube formation ability of HUVECs in each group using an inverted microscope (Figure 2H). The number of connection points (Figure 2I) and total branch length (Figure 2J) were quantified. The AS-IV@HdECM hydrogel group exhibited an average number of 53 connection points, significantly higher than the 12, 23, and 39 connection points observed in the MI group, HdECM hydrogel group, and AS-IV group, respectively. A similar trend was observed in total branch length. Overall, these results highlight the superior angiogenic potential of the AS-IV@HdECM hydrogel, which outperformed the MI group, HdECM hydrogel group, and AS-IV group in promoting both angiogenesis and wound healing.

Finally, apoptotic cell death was assessed by TUNEL staining, which revealed that OGD treatment significantly induced apoptosis in HUVECs (Figure 2K). In contrast, all three treatment groups-HdECM hydrogel, AS-IV, and AS-IV@HdECM hydrogel-markedly reduced the level of apoptosis (Figure 2L). These results underscore the protective effects of the treatments against ischemia-induced endothelial cell damage.

Taken together, our results demonstrate that the AS-IV@HdECM hydrogel, along with HdECM hydrogel and AS-IV, mitigates myocardial ischemia and hypoxia-induced injury by promoting angiogenesis, enhancing endothelial cell migration and suppressing apoptosis. These multifaceted therapeutic effects not only supports vascular repair but also fosters tissue regeneration, positioning the AS-IV@HdECM hydrogel system as a promising strategy for treating ischemic diseases.

### 3.3 AS-IV@HdECM significantly improves cardiac function after acute myocardial infarction

#### 3.3.1 AS-IV@HdECM upregulates CD31 and α-SMA, and reduces cardiomyocyte apoptosis

**FIGURE 3:**
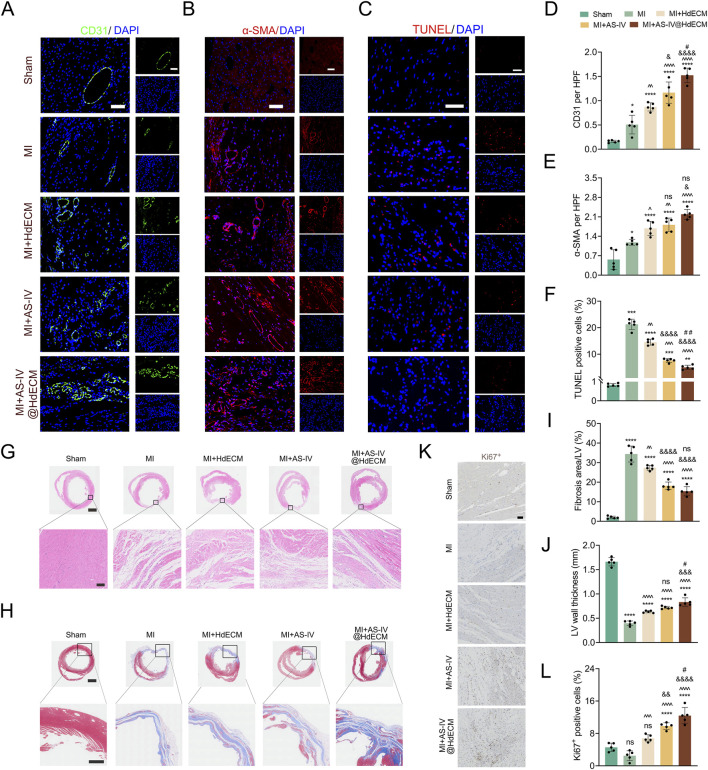
Morphological and histological evaluation of the therapeutic efficacy of AS-IV@HdECM hydrogel. Representative immunofluorescence images of vascular endothelium CD31 [**(A)**, green] and small arteries α-SMA [**(B)**, red] (scale bar:100 μm), alongside quantitative analysis of vascular conditions in the infarcted area (**(D,E)**, n = 5). **(C)** TUNEL staining (red) (scale bar:50 μm) and **(F)** quantitative evaluation of myocardial cell injury in the infarcted heart border zone (n = 5); DAPI was used to label the cell nuclei. **(G)** Images showing myocardial morphological changes (scale bars:2 mm and 100 μm). **(H)** Masson staining images (scale bars:2 mm and 500 μm), with corresponding quantitative analysis of **(I)** myocardial fibrosis and **(J)** ventricular wall thickness (n = 5). **(K)** Cell proliferation (scale bar:50 μm) and **(L)** quantitative analysis (n = 5). All bar graphs display individual data points.

To investigate the therapeutic effect of AS-IV@HdECM hydrogel in a more physiologically relevant model, we conducted animal studies using a MI rat model. The drug was carefully administered into the pericardial cavity of the rats, and after 28 days of treatment, we preformed immunofluorescence analysis on heart tissue to evaluate the distribution and density of microvessels and small arteries within the treated myocardial tissue (Chen et al., 2023; Luo et al., 2023). The results showed a significant upregulation of CD31, a marker of endothelial cells, and α-SMA, a marker of smooth muscle cells, in the treatment groups compared to the MI model group. Notably, the AS-IV@HdECM group exhibited the most pronounced increase in the expression of these markers, indicating a strong promotion of vascular formation (Figures 3A,B,D,E). This suggests that the AS-IV@HdECM treatment not only promotes endothelial cell proliferation and migration but also aids in the recruitment of smooth muscle cells, thus supporting the stabilization of newly formed blood vessels. In addition, TUNEL immunofluorescence analysis revealed a significant attenuation in cardiomyocyte apoptosis in the AS-IV@HdECM group, indicating that the treatment effectively mitigated myocardial cell damage (Figures 3C,F). Given the critical role of vascular regeneration in myocardial protection, our system improves local blood perfusion, delivers essential nutrients and oxygen to the infarcted myocardium, and thus facilitates myocardial repair.

#### 3.3.2 Evaluation of pathological changes in myocardial tissue

Regarding cardiac morphology and structure,H&E staining revealed that myocardial cells in the Sham group were well-organized, exhibiting uniform staining and minimal infiltration of inflammatory cell. In contrast, the MI group displayed disorganized myocardial cell structures, with indistinct cell boundaries, widened intercellular spaces, and extensive inflammatory cell infiltration. Following 28 days of treatment with AS-IV, HdECM or AS-IV@HdECM, myocardial injury and inflammation were markedly alleviated. The AS-IV@HdECM group exhibited the most pronounced improvement in myocardial architecture, with reduced inflammation and better organization (Figure 3G).

Masson trichrome staining further revealed that, in the Sham group, the myocardium exhibited a bright red coloration with uniform cytoplasmic staining. However, in the MI group, there was substantial accumulation of blue collagen around the infarction area, along with thinning of the ventricular wall (Figure 3H). In all three treatment groups, collagen density in myocardial tissue was notably reduced, with a significant decrease in collagen deposition and thickening of the ventricular wall (Figures 3I,J). Quantitative analysis revealed that the infarct size in the AS-IV@HdECM group was reduced to 15.4%, the most significant reduction among the treatment groups.

Immunohistochemical staining for Ki67+ proliferating cells further demonstrated prominent cell proliferation in the AS-IV@HdECM-treated group (Figures 3K,L). These findings indicate that the AS-IV@HdECM treatment not only enhances cell proliferation in the ischemic infarct area but also ameliorates myocardial injury, and attenuates fibrosis.

#### 3.3.3 Sustained drug release and hydrogel metabolism within the pericardial cavity

**FIGURE 4:**
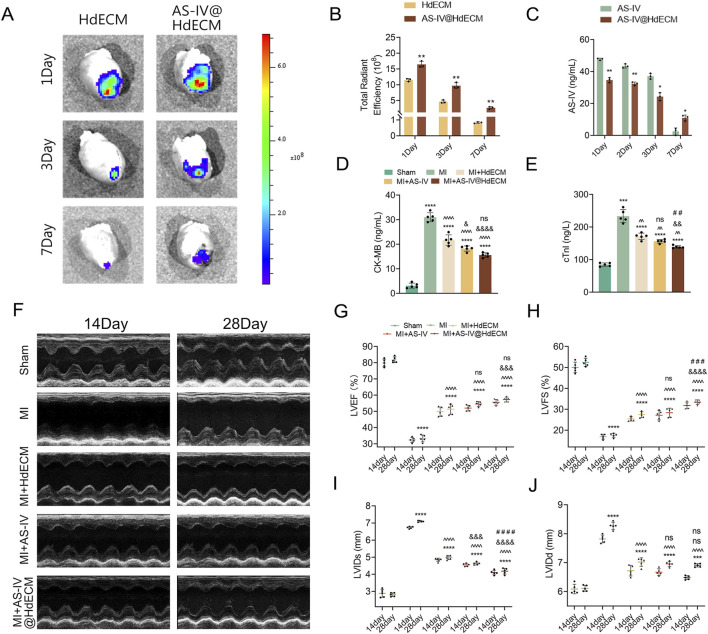
*In vivo* sustained release and cardiac function assessment. **(A)** Fluorescence images of HdECM, and AS-IV@HdECM (n = 3) injected in intrapericardial cavity (IPC) and **(B)** quantitative analysis of fluorescence signal. **(C)** Time-dependent concentration of released AS-IV in the serum of the treatment groups AS-IV and AS-IV@HdECM (n = 3). **(D,E)** Bar graphs depicting CK-MB and cTnI levels in each group (n = 5). **(F)** Representative echocardiographic images of different groups at 14 and 28 days post the treatment. Assessment of cardiac function parameters: **(G)** Left ventricular ejection fraction (LVEF), **(H)** Left ventricular fractional shortening (LVFS), **(I)** left ventricular internal diameter at end-systole (LVIDs), and **(J)** left ventricular internal diameter at end-diastole (LVIDd) (n = 5). All bar graphs include individual data points.

To assess the metabolism behavior of the hydrogel system *in vivo*, HdECM, and AS-IV@HdECM hydrogels were labeled with rhodamine fluorescent dye and administered into the intrapericardial cavity of rats following MI modeling. Fluorescence images were captured at 1, 3, and 7 days post-administration to monitor the hydrogel’s retention and degradation (Figure 4A). Quantitative analysis revealed a gradual decline in fluorescence intensity over time for all two systems (Figure 4B). Notably, HdECM when administered alone exhibited the fastest reduction in fluorescence, indicating rapid degradation. In contrast, AS-IV@HdECM hydrogel showed a more stable and prolonged presence at the injection site, maintaining stronger fluorescence signals throughout the study period compared to HdECM alone. As mentioned in previous *in vitro* experiments, the AS-IV@HdECM hydrogel displayed a more compact porous structure and slower degradation kinetics than the HdECM hydrogel, which likely contributed to the higher retention observed in the pericardial cavity. Furthermore, based on the thermal sensitivity and tissue adhesiveness shown in Figures 1A,B, the AS-IV@HdECM hydrogel can form a functional, patch-like depot area within the pericardial environment, slowly releasing AS-IV locally to support the regeneration of local microvessels.

To further evaluate the systemic distribution of AS-IV, plasma samples were collected from rats at 1, 3, and 7 days post-administration to quantify AS-IV levels in the circulation for both the AS-IV and AS-IV@HdECM hydrogel groups. In the first 3 days, AS-IV concentrations in palsma was lower for the AS-IV@HdECM hydrogel group compared to AS-IV group alone, consistent with the retention of AS-IV by HdECM hydrogel in the pericardic area (Figure 4C). Interestinly, AS-IV concentrations remained over 20 ng/mL in the plasma 3 days post-administration, whereas previous studies have reported no detectable levels of AS-IV after the same duration when administered orally. This underscores the advantage of local administration for providing extended therapeutic effects. Furthermore, the HdECM hydrogel prolonged drug retention for at least 7 days, as evidenced by the continued presence of AS-IV in the plasma for AS-IV@HdECM hydrogel, while AS-IV was undetectable in the plasma for AS-IV group. These findings highlight the potential of the AS-IV@HdECM hydrogel to deliver sustained therapeutic effects on the heart over an extended period.

#### 3.3.4 Evaluation of cardiac function

Plasma levels of CK-MB and cTnI serve as critical biomarkers of myocardial injury. Elevated levels of both CK-MB and cTnI typically indicate myocardial damage. These biomarkers were measured on the second day following intrapericardial cavity (IPC) injections (Figures 4D,E). As anticipated, both CK-MB and cTnI levels were significantly elevated in the MI model, reflecting acute myocardial injury, which typically lasts approximately 48 h. However, treatment with HdECM, AS-IV, and AS-IV@HdECM resulted in a noticeable reduction in myocardial enzyme levels, with the combined AS-IV@HdECM formulation showing the most substantial decrease in both CK-MB and cTnI. This suggests a protective effect on the heart.

Subsequently, on days 14 and 28 post-myocardial infarction, echocardiography was conducted to evaluate the impact of the treatments on cardiac function. Several key parameters, including LVEF, LVFS, LVIDd and LVIDs were measured. Compared to the sham operation group, the MI group exhibited notable pathological changes, such as significant thinning of the left ventricular anterior wall (Figure 4F) and a dramatic decline in both LVEF and LVFS, which dropped to 32.3% and 16.9%, respectively. Concurrently, LVIDd and LVIDs were significantly increased, reflecting impaired cardiac systolic and diastolic functions and confirming the successful establishment of the MI model. Data analysis (Figures 4G–J) revealed that all treatment groups exhibited significant improvements in cardiac function, as evidenced by enhanced EF and FS, and marked reductions in LVIDd and LVIDs. In particular, the superior therapeutic efficacy of the AS-IV@HdECM group over other treatment groups highlights its potential as a promising intervention for cardiac repair post-MI.

#### 3.3.5 Evaluation of drug toxicity, side effects, and inflammatory responses

H&E staining of tissue sections from the liver, spleen, lung, and kidney was performed to evaluate cellular morphology and potential histopathological changes (Wang K. et al., 2023). The cellular architecture of all tissues appeared normal, with uniform staining and an even distribution of nuclei and cytoplasm, suggesting no significant damage or abnormalities. The boundaries between the different tissue components were clearly delineated, indicating preserved tissue integrity (Supplementary Figure S2).

To further assess organ function, biochemical markers indicative of liver and kidney health were quantified. Specifically, levels of ALT, AST,UA and Scr were measured, all of which showed no significant deviations from normal ranges. The three therapeutic drug groups exerted minimal toxicity on the rat organs and exhibited a low potential for accumulation within the body (Supplementary Figure S3).

As the peak period of acute inflammatory response occurs 24–48 h after infarction, on the second day post-operation, we further investigated the plasma levels of pro-inflammatory cytokines, including IL-6, IL-1β, and TNF-α. The data revealed that rats in the MI model group had significantly elevated levels of these cytokines compared to the control group, indicating the presence of a cytokine storm associated with MI. However, all three treatment groups effectively reduced pro-inflammatory cytokine levels (Supplementary Figure S4). These findings suggest that the therapeutic effects of the drugs may be attributed not only to their direct action on myocardial injury but also to their ability to mitigate the inflammatory cascade triggered by the MI-induced damage. Moreover, the results indicate that the metabolic byproducts of these drugs do not cause any discernible adverse reactions, further supporting their safety profile *in vivo*.

### 3.4 Analyzing potential action pathways

#### 3.4.1 Network pharmacology analysis

**FIGURE 5:**
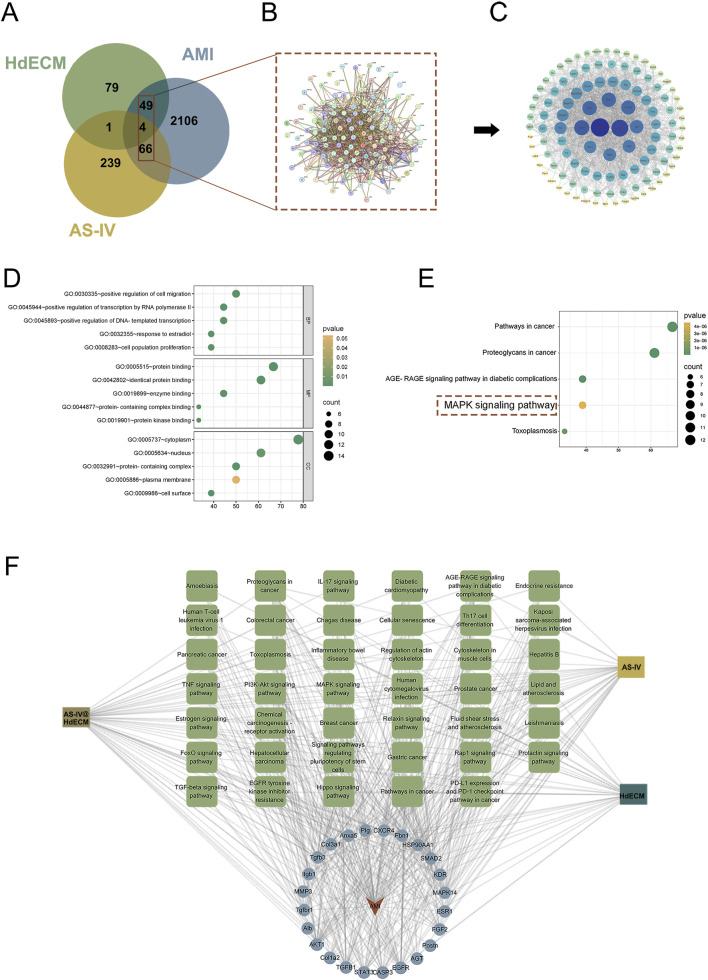
Network pharmacology analysis of HdECM, AS-IV, and AS-IV@HdECM in the treatment of MI. **(A)** Venn diagram illustrating overlapping targets among HdECM, AS-IV, and MI. **(B)** PPI network of the intersecting genes. **(C)** Core targets identified from the intersecting genes (color intensity indicates the degree of centrality, with darker blue representing higher centrality). **(D)** GO enrichment analysis of the top five biological processes associated with the target genes. **(E)** The top five KEGG enrichment pathways. **(F)** Network diagram illustrating the interactions among drugs, disease, targets, and pathways.

A comprehensive proteomic analysis of the primary components of HdECM, combined with an extensive database screening (Yang et al., 2021), identified 133 targets associated with HdECM, 310 targets related to AS-IV, and 2225 targets linked to MI were identified. To uncover potential therapeutic targets for MI, a Venn diagram was constructed to visualize the overlapping genes across these datasets. The analysis revealed that HdECM shared 53 targets, AS-IV shared 70 targets, and the combined AS-IV@HdECM group exhibited 119 overlapping targets. These 119 shared targets were identified as promising therapeutic candidates for MI (Figure 5A).

To further validate the significance of these targets, the 119 common targets were input into the STRING database for protein-protein interaction (PPI) network topology analysis (Figure 5B). The resulting PPI network was reconstructed using Cytoscape, which enabled the identification of the top 18 core based on a Degree value threshold of ≥30 (Figure 5C). These 18 core targets are considered key players in the molecular mechanisms underlying MI.

To gain deeper insights into the biological processes and signaling pathways potentially modulated by these 18 core targets in the context of MI, Gene Ontology (GO) annotation and functional enrichment analysis were performed using the DAVID and Microbiomics platforms. GO biological process (BP) analysis revealed that the target genes were predominantly enriched in processes such as cell migration, DNA transcription, and regulation of RNA polymerase II-mediated signal transduction. Cellular component (CC) analysis highlighted cellular structures like the nucleus, cytoplasm, and ion channel lumen, while molecular function (MF) analysis identified key molecular functions including protein binding, enzyme binding, and protein phosphatase binding (Figure 5D; Supplementary Figure S6).

Furthermore, Kyoto Encyclopedia of Genes and Genomes (KEGG) pathway enrichment analysis identified the top 30 pathways associated with MI treatment, revealing four major mechanisms: vascular regeneration pathways (such as the MAPK and PI3K-Akt pathways), inflammation and immune response-related pathways (e.g., TNF and Rap1 signaling), cell death-related pathways (e.g., apoptosis and cellular senescence), and endocrine resistance-related pathways (e.g., lipid and glucose metabolism) (Figure 5E; Supplementary Figure S7). To offer a more intuitive representation, the drug-disease-target-pathway interaction network (Figure 5F) was constructed, illustrating the intricate relationships between therapeutic agents, MI, and the identified pathways, thereby shedding light on the multifaceted mechanism involved in MI treatment.

#### 3.4.2 Verification of drug-target interactions by molecular docking

**FIGURE 6:**
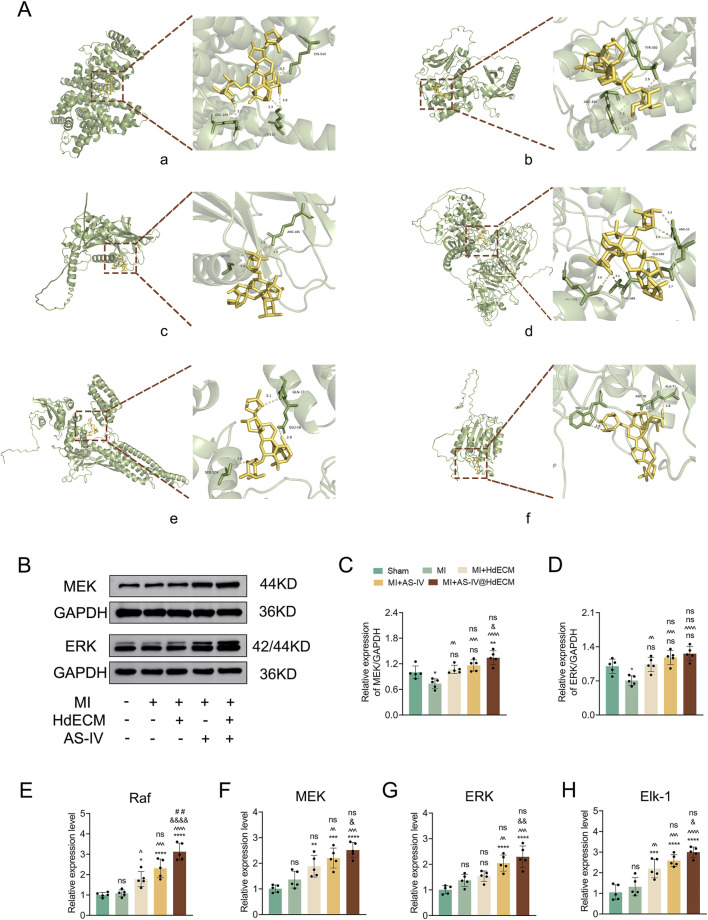
Effects of molecular docking and target expression analysis *in vitro*. **(A)** Visualization of molecular docking results: **(a)** ALB, **(b)** AKT1, **(c)** TGFB1, **(d)** EGFR, **(e)** STAT3, **(f)** CASP3. **(B–D)** Representative Western blot images and quantitative analysis of protein expression levels in the pathway. **(E–H)** Quantitative analysis of relative gene expression levels in the pathway.

Molecular docking was employed to assess the binding affinity between astragaloside and the proteins expressed by six key targets: ALB, AKT1, TGFB1, EGFR, STAT3 and CASP3. The results are visualized in Figure 6A. The binding energies for these interactions were determined to be −7.4, −7.2, −7.1, −8.1, −7.3, and −7.2 kcal/mol, respectively. A binding affinity of ≤ −5.0 kcal/mol is generally indicative of a strong interaction between the ligand and receptor. Lower (more negative) binding energies suggests a more stable ligand-receptor complex (Jiang et al., 2024; Yang et al., 2023). These findings demonstratethat astragaloside exhibits strong binding affinity toward all six targets, with EGFR showing the highest affinity.

#### 3.4.3 *In vitro* verification of the underlying mechanism

Building on the optimal binding energy of EGFR observed in molecular docking and the MAPK signaling pathway identified in the KEGG analysis, we further investigated the role of EGFR in activating the classical Raf-MEK-ERK cascade, a crucial branch of the MAPK pathway (Ullah et al., 2022; Wu et al., 2024). We measured the expression levels of MEK and ERK proteins in infarcted myocardial tissue and performed semi-quantitative analysis via Western blotting (Figures 6B–D). Additionally, q-PCR was employed to evaluate the gene expression levels of MEK, ERK, the upstream regulator Raf, and the downstream effector Elk-1 (Kaur et al., 2010; Zhang and Liu, 2002). The results (Figures 6E–H) revealed that AS-IV@HdECM significantly upregulated the expression of these proteins and genes, indicating its potential to protect endothelial cells, promote angiogenesis and enhance microcirculation. Additionally,the results showed that the EGFR pathway expression in these control groups was significantly lower than that in the AS-IV-treated groups, even though the control groups still maintained basic cell activity. This indicates that the upregulation of the EGFR pathway is not merely a passive downstream consequence of enhanced cell activity, but is specifically induced by AS-IV.

## 4 Conclusion

In conclusion, the bioactive AS-IV@HdECM hydrogel presents a promising therapeutic strategy for enhancing myocardial repair following MI. By encapsulating AS-IV with in a heart-derived extracellular matrix hydrogel, this system effectively improves the bioavailability and controlled release of AS-IV within the myocardium. Unlike the traditional intramyocardial injection method, intrapericardial administration enables the hydrogel to form a patch-like depot area on the surface of the heart, allowing for long-term retention within the heart while causing minimal damage to the tissue. Through the combination of *in vitro* and *in vivo* studies, as well as the integration of proteomics and network pharmacology analysis, we demonstrated that the therapeutic effect of AS-IV@HdECM is related to the activation of the EGFR/Raf-MEK-ERK signaling pathway. This activation promotes angiogenesis, reduces fibrosis, and improves cardiac function after myocardial infarction. A limitation of the present study is that specific inhibitor experiments were not performed to further verify the causal relationship between EGFR pathway activation and the observed therapeutic effects, which will be addressed in our future mechanistic investigations. This approach combines the principles of TCM with modern bioengineering, offering a novel, effective method for MI treatment. These findings lay a strong foundation for the potential transformation in improving cardiac function and outcomes post-MI.

## Data Availability

The raw data supporting the conclusions of this article are openly available in Figshare and made available by the authors, without undue reservation.

## References

[B1] Bianconi V. Sahebkar A. Kovanen P. Bagaglia F. Ricciuti B. Calabrò P. (2018). Endothelial and cardiac progenitor cells for cardiovascular repair: a controversial paradigm in cell therapy. Pharmacol. and Ther. 181, 156–168. 10.1016/j.pharmthera.2017.08.004 28827151

[B2] Cabac‐Pogorevici I. Muk B. Rustamova Y. Kalogeropoulos A. Tzeis S. Vardas P. (2020). Ischaemic cardiomyopathy. Pathophysiological insights, diagnostic management and the roles of revascularisation and device treatment. Gaps and dilemmas in the era of advanced technology. Eur. J Heart Fail 22, 789–799. 10.1002/ejhf.1747 32020756

[B3] Chang Y. Sun Y. Li J. Zhang Q.-H. Guo X.-R. Zhang B. (2012). The experimental study of Astragalus membranaceus on meridian tropsim: the distribution study of astragaloside IV in rat tissues. J. Chromatogr. B 911, 71–75. 10.1016/j.jchromb.2012.10.024 23217309

[B4] Chaudhuri O. Cooper-White J. Janmey P. A. Mooney D. J. Shenoy V. B. (2020). Effects of extracellular matrix viscoelasticity on cellular behaviour. Nature 584, 535–546. 10.1038/s41586-020-2612-2 32848221 PMC7676152

[B5] Chen A. Mesfin J. M. Gianneschi N. C. Christman K. L. (2023). Intravascularly deliverable biomaterial platforms for tissue repair and regeneration post‐myocardial infarction. Adv. Mater. 36, 2300603. 10.1002/adma.202300603 36989469 PMC10539487

[B6] Cheng S. Zhang X. Feng Q. Chen J. Shen L. Yu P. (2019). Astragaloside IV exerts angiogenesis and cardioprotection after myocardial infarction via regulating PTEN/PI3K/Akt signaling pathway. Life Sci. 227, 82–93. 10.1016/j.lfs.2019.04.040 31004658

[B7] Gil-Cabrerizo P. Scacchetti I. Garbayo E. Blanco-Prieto M. J. (2023). Cardiac tissue engineering for myocardial infarction treatment. Eur. J. Pharm. Sci. 185, 106439. 10.1016/j.ejps.2023.106439 37003408

[B8] Gómez-Cid L. López-Donaire M. L. Velasco D. Marín V. González M. I. Salinas B. (2021). Cardiac extracellular matrix hydrogel enriched with polyethylene glycol presents improved gelation time and increased On-Target site retention of extracellular vesicles. IJMS 22, 9226. 10.3390/ijms22179226 34502146 PMC8431142

[B9] Grune J. Lewis A. J. M. Yamazoe M. Hulsmans M. Rohde D. Xiao L. (2022). Neutrophils incite and macrophages avert electrical storm after myocardial infarction. Nat. Cardiovasc Res. 1, 649–664. 10.1038/s44161-022-00094-w 36034743 PMC9410341

[B10] Jacot J. G. Martin J. C. Hunt D. L. (2010). Mechanobiology of cardiomyocyte development. J. Biomechanics 43, 93–98. 10.1016/j.jbiomech.2009.09.014 19819458 PMC2813357

[B11] Jiang B. Liu J. Qu Z. Wang Y. Wang Y. Li Z. (2024). Mechanism of dihydroartemisinin in the treatment of ischaemia/reperfusion-induced acute kidney injury via network pharmacology, molecular dynamics simulation and experiments. Int. Immunopharmacol. 144, 113705. 10.1016/j.intimp.2024.113705 39626534

[B12] Kaur J. Adya R. Tan B. K. Chen J. Randeva H. S. (2010). Identification of chemerin receptor (ChemR23) in human endothelial cells: chemerin-induced endothelial angiogenesis. Biochem. Biophysical Res. Commun. 391, 1762–1768. 10.1016/j.bbrc.2009.12.150 20044979

[B13] Li J. Lv Y. Zhu D. Mei X. Huang K. Wang X. (2022). Intrapericardial hydrogel injection generates high cell retention and augments therapeutic effects of mesenchymal stem cells in myocardial infarction. Chem. Eng. J. 427, 131581. 10.1016/j.cej.2021.131581

[B14] Luo L. Li Y. Bao Z. Zhu D. Chen G. Li W. (2023). Pericardial delivery of SDF‐1 *α* puerarin hydrogel promotes heart repair and electrical coupling. Adv. Mater. 36, 2302686. 10.1002/adma.202302686 37665792

[B15] Prabhu S. D. Frangogiannis N. G. (2016). The biological basis for cardiac repair after myocardial infarction: from inflammation to fibrosis. Circulation Res. 119, 91–112. 10.1161/CIRCRESAHA.116.303577 27340270 PMC4922528

[B16] Song Y. You Y. Xu X. Lu J. Huang X. Zhang J. (2023). Adipose‐derived mesenchymal stem cell‐derived exosomes biopotentiated extracellular matrix hydrogels accelerate diabetic wound healing and skin regeneration. Adv. Sci. 10, 2304023. 10.1002/advs.202304023 37712174 PMC10602544

[B17] Spang M. T. Middleton R. Diaz M. Hunter J. Mesfin J. Banka A. (2022). Intravascularly infused extracellular matrix as a biomaterial for targeting and treating inflamed tissues. Nat. Biomed. Eng. 7, 94–109. 10.1038/s41551-022-00964-5 36581694 PMC10166066

[B18] Sui Y.-B. Wang Y. Liu L. Liu F. Zhang Y.-Q. (2019). Astragaloside IV alleviates heart failure by promoting angiogenesis through the JAK-STAT3 pathway. Pharm. Biol. 57, 48–54. 10.1080/13880209.2019.1569697 30905241 PMC8871603

[B19] Sun C. Zeng G. Wang T. Ren H. An H. Lian C. (2021). Astragaloside IV ameliorates myocardial infarction induced apoptosis and restores cardiac function. Front. Cell Dev. Biol. 9, 671255. 10.3389/fcell.2021.671255 34395418 PMC8358605

[B20] Traverse J. H. Henry T. D. Dib N. Patel A. N. Pepine C. Schaer G. L. (2019). First-in-Man study of a cardiac extracellular matrix hydrogel in early and late myocardial infarction patients. JACC Basic Transl. Sci. 4, 659–669. 10.1016/j.jacbts.2019.07.012 31709316 PMC6834965

[B21] Ullah R. Yin Q. Snell A. H. Wan L. (2022). RAF-MEK-ERK pathway in cancer evolution and treatment. Seminars Cancer Biol. 85, 123–154. 10.1016/j.semcancer.2021.05.010 33992782

[B22] Ungerleider J. L. Johnson T. D. Rao N. Christman K. L. (2015). Fabrication and characterization of injectable hydrogels derived from decellularized skeletal and cardiac muscle. Methods 84 (4.67), 53–59. 10.1016/j.ymeth.2015.03.024 25843605 PMC4526417

[B23] Virani S. S. Alonso A. Aparicio H. J. Benjamin E. J. Bittencourt M. S. Callaway C. W. (2021). Heart disease and stroke Statistics—2021 update: a report from the American heart association. Circulation 143, e254–e743. 10.1161/CIR.0000000000000950 33501848 PMC13036842

[B24] Wang X. Ansari A. Pierre V. Young K. Kothapalli C. R. Von Recum H. A. (2022). Injectable extracellular matrix microparticles promote heart regeneration in mice with post‐ischemic heart injury. Adv. Healthc. Mater. 11, 2102265. 10.1002/adhm.202102265 35118812 PMC9035118

[B25] Wang B. Qinglai T. Yang Q. Li M. Zeng S. Yang X. (2023a). Functional acellular matrix for tissue repair. Mater. Today Bio 18, 100530. 10.1016/j.mtbio.2022.100530 36601535 PMC9806685

[B26] Wang K. Zhu K. Zhu Z. Shao F. Qian R. Wang C. (2023b). Triptolide with hepatotoxicity and nephrotoxicity used in local delivery treatment of myocardial infarction by thermosensitive hydrogel. J. Nanobiotechnol 21, 227. 10.1186/s12951-023-01980-6 37461079 PMC10351172

[B27] Wu M.-Y. Yiang G.-T. Liao W.-T. Tsai A. P.-Y. Cheng Y.-L. Cheng P.-W. (2018). Current mechanistic concepts in ischemia and reperfusion injury. Cell Physiol. Biochem. 46, 1650–1667. 10.1159/000489241 29694958

[B28] Wu X. Reboll M. R. Korf-Klingebiel M. Wollert K. C. (2021). Angiogenesis after acute myocardial infarction. Cardiovasc. Res. 117, 1257–1273. 10.1093/cvr/cvaa287 33063086

[B29] Wu Y. Fang Y. Li Y. Au R. Cheng C. Li W. (2024). A network pharmacology approach and experimental validation to investigate the anticancer mechanism of qi-qin-hu-chang formula against colitis-associated colorectal cancer through induction of apoptosis *via* JNK/p38 MAPK signaling pathway. J. Ethnopharmacol. 319, 117323. 10.1016/j.jep.2023.117323 37852337

[B30] Xu Z. Zhou H. Zhang Y. Cheng Z. Wan M. Qin W. (2023). Recent pharmacological advances in the treatment of cardiovascular events with astragaloside IV. Biomed. & Pharmacother. 168, 115752. 10.1016/j.biopha.2023.115752 37875045

[B31] Yang X. Chen S. Chen J. Liu Y. Bai Y. Yin S. (2021). The different effect of decellularized myocardial matrix hydrogel and decellularized small intestinal submucosa matrix hydrogel on cardiomyocytes and ischemic heart. Appl. Sci. 11, 7768. 10.3390/app11177768

[B32] Yang A.-Y. Liu H.-L. Yang Y.-F. (2023). Study on the mechanism of action of Scutellaria barbata on hepatocellular carcinoma based on network pharmacology and bioinformatics. Front. Pharmacol. 13, 1072547. 10.3389/fphar.2022.1072547 36699068 PMC9869961

[B33] Yin B. Hou X. Lu M. (2019). Astragaloside IV attenuates myocardial ischemia/reperfusion injury in rats *via* inhibition of calcium-sensing receptor-mediated apoptotic signaling pathways. Acta Pharmacol. Sin. 40, 599–607. 10.1038/s41401-018-0082-y 30030530 PMC6786296

[B34] Zhang W. Liu H. T. (2002). MAPK signal pathways in the regulation of cell proliferation in mammalian cells. Cell Res. 12, 9–18. 10.1038/sj.cr.7290105 11942415

[B35] Zhang W.-D. Zhang C. Liu R.-H. Li H.-L. Zhang J.-T. Mao C. (2006). Preclinical pharmacokinetics and tissue distribution of a natural cardioprotective agent astragaloside IV in rats and dogs. Life Sci. 79, 808–815. 10.1016/j.lfs.2006.02.032 16564551

[B36] Zhang W. Du A. Liu S. Lv M. Chen S. (2021). Research progress in decellularized extracellular matrix-derived hydrogels. Regen. Ther. 18, 88–96. 10.1016/j.reth.2021.04.002 34095366 PMC8142036

[B37] Zhang W. Zhang L. Zhou H. Li C. Shao C. He Y. (2022a). Astragaloside IV alleviates infarction induced cardiomyocyte injury by improving mitochondrial morphology and function. Front. Cardiovasc. Med. 9, 810541. 10.3389/fcvm.2022.810541 35265681 PMC8899080

[B38] Zhang X. Qu H. Yang T. Liu Q. Zhou H. (2022b). Astragaloside IV attenuate MI-induced myocardial fibrosis and cardiac remodeling by inhibiting ROS/caspase-1/GSDMD signaling pathway. Cell Cycle 21, 2309–2322. 10.1080/15384101.2022.2093598 35770948 PMC9586672

