# Supplementary material for: A bioactive heart-derived ECM hydrogel potentiates Astragaloside IV–mediated microvascular regeneration
Source: Front Bioeng Biotechnol. 2026 Apr 8;14:1800990. doi: 10.3389/fbioe.2026.1800990 (PMC13099876; doi:10.3389/fbioe.2026.1800990)
Supplement: Supplementary file 1 [file Supplementaryfile1.docx]

The following files are available free of charge


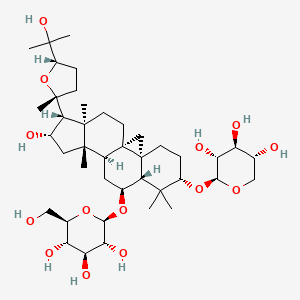


Fig. S1 The chemical structure of AS-IV (https://pubchem.ncbi.nlm.nih.gov/).

| **β-Actin** | **FORWARD** | **5’CCACTGCCGCATCCTCTT3’** |
| --- | --- | --- |
|  | **REVERSE** | **5’GCATCGGAACCGCTCATT3’** |
| **RAF** | **FORWARD** | **5’ATGGCTCCAGTTGCATCTCC3’** |
|  | **REVERSE** | **5’CATAAGGCAGTCGTGCAAGC3’** |
| **MEK** | **FORWARD** | **5’ATGCCCAAGAAGAAGCCGAC3’** |
|  | **REVERSE** | **5’ATGGCTTGTGGGAGACCTTG3’** |
| **ERK** | **FORWARD** | **5’TGCAGAGATCGTCAACACCC3’** |
|  | **REVERSE** | **5’TCATCCACGGTGGTAAAGGC3’** |
| **Elk-1** | **FORWARD** | **5’CTATGGGGACTGCGCAAGAA3’** |
|  | **REVERSE** | **5’CTACGGCCGAGGTTACAGAC3’** |

Table S1 **The primer sequences.**


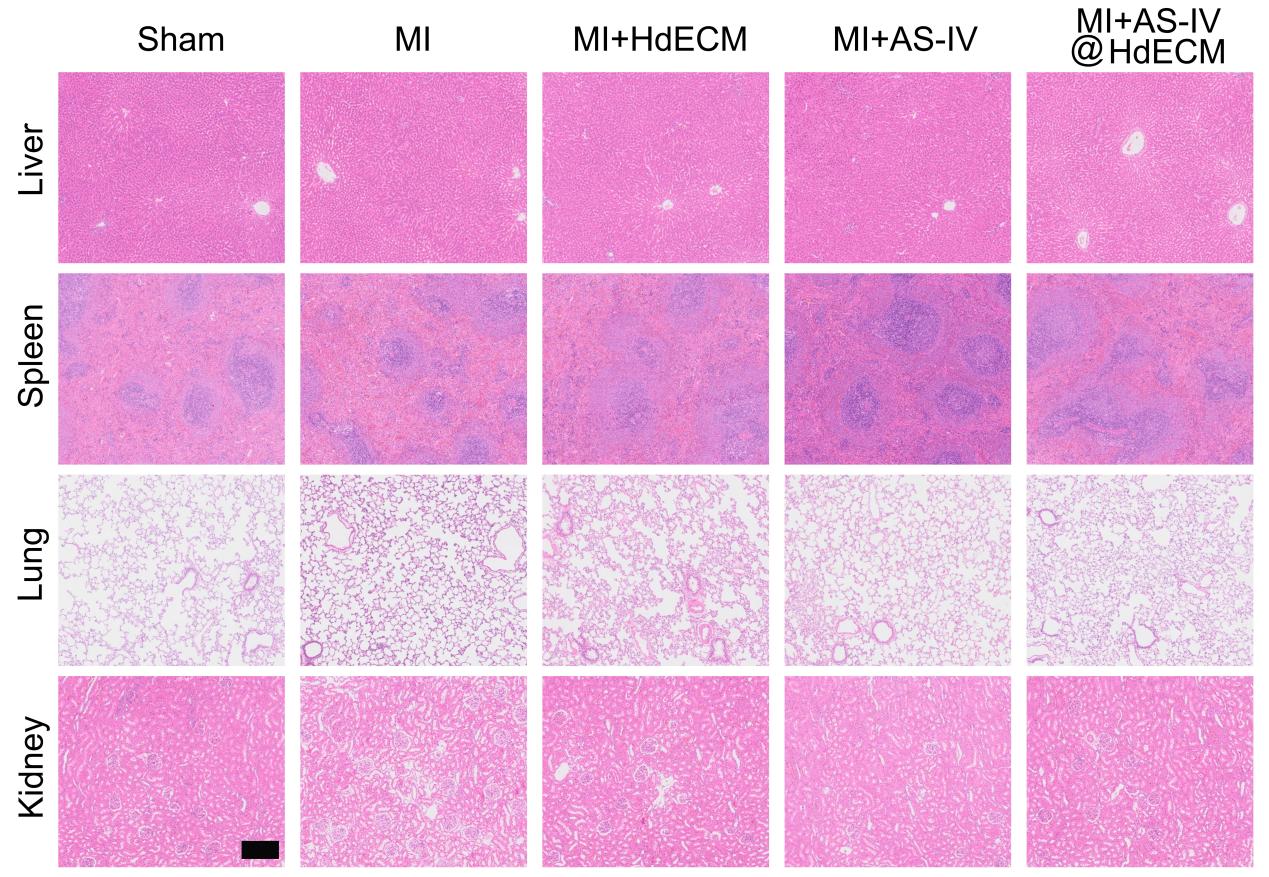


Fig. S2 **H&E staining of the major organs, including the liver, spleen, lung, and kidney (scale bar:200μm).**


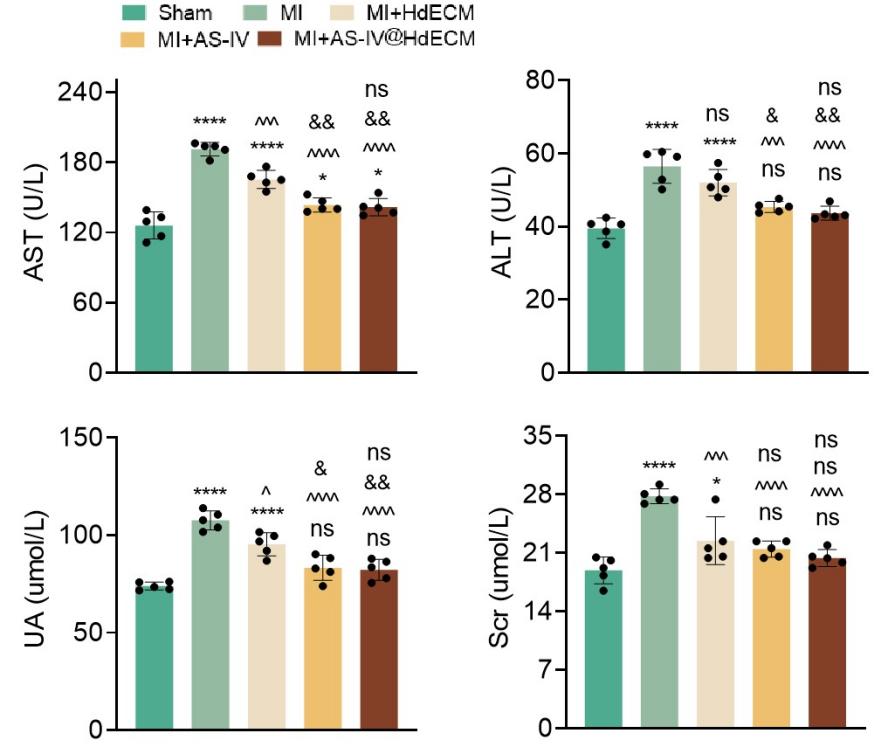


Fig. S3 **The effects of 28 days drug administration on serum levels of AST,ALT,UA and Scr.**


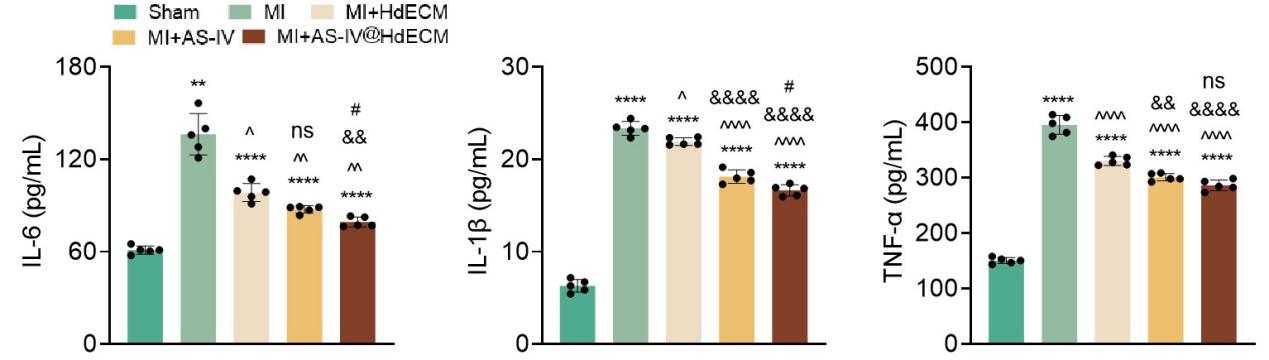


**Fig. S4 The impact of different drug administration groups on serum levels of IL-6, IL-1β and TNF-α on the second day post-myocardial infarction.**

| FBN1 | PPARA | COL1A2 | CSRP3 | NPR1 | COL5A2 |
| --- | --- | --- | --- | --- | --- |
| MIA | TGM2 | ELN | ATXN2 | ITGB1 | ANGPTL3 |
| LDB3 | FGA | COL4A1 | PLG | COL4A2 | MYH6 |
| FBLN5 | TTN | TCP1 | THBS4 | ATP2B2 | LTBP4 |
| GYS1 | VCAN | ITGB2 | POSTN | COL3A1 | TUBB |
| LAMA2 | FABP3 | SDHA | TNNT2 | C3 | CRP |
| CKM | SMAD2 | SMAD4 | TGFB1 | TGFB3 | TGFB2 |
| TGFBR3 | FOS | MSTN | INHA | VEGFB | SPARC |
| HBEGF | ALB | TGFBR2 | TGFBR1 | EGFR | TFRC |
| CASP3 | SOD2 | ESR1 | F2 | LGALS3 | AKT1 |
| MAPK1 | CTSB | ANXA5 | MMP13 | GSR | CCNA2 |
| BCHE | MAPK14 | DPP4 | FGF2 | MMP3 | PIK3CG |
| PLA2G1B | PTPN11 | STAT3 | VEGFA | CXCR4 | PARP1 |
| KDR | HPSE | ADRA2B | CHI3L1 | MAPK8 | PTGS1 |
| CTSD | FGF1 | GSTP1 | TTR | ADRA1A | INS |
| AR | MTAP | PIM1 | PPIA | BMP2 | ADH1B |
| CYP2D6 | DRD1 | EPHB4 | AKT2 | FCAR | GC |
| ANG | HSP90AA1 | APOA2 | MET | MSRA | P4HB |
| PPID | MAPK10 | PRNP | PSEN1 | PTPRA | CFB |
| ADAM17 | TTPA | CA2 | CDK1 | ACTA1 |  |

Table S2 The 119 interaction genes between AS-IV,HdECM and MI related genes.


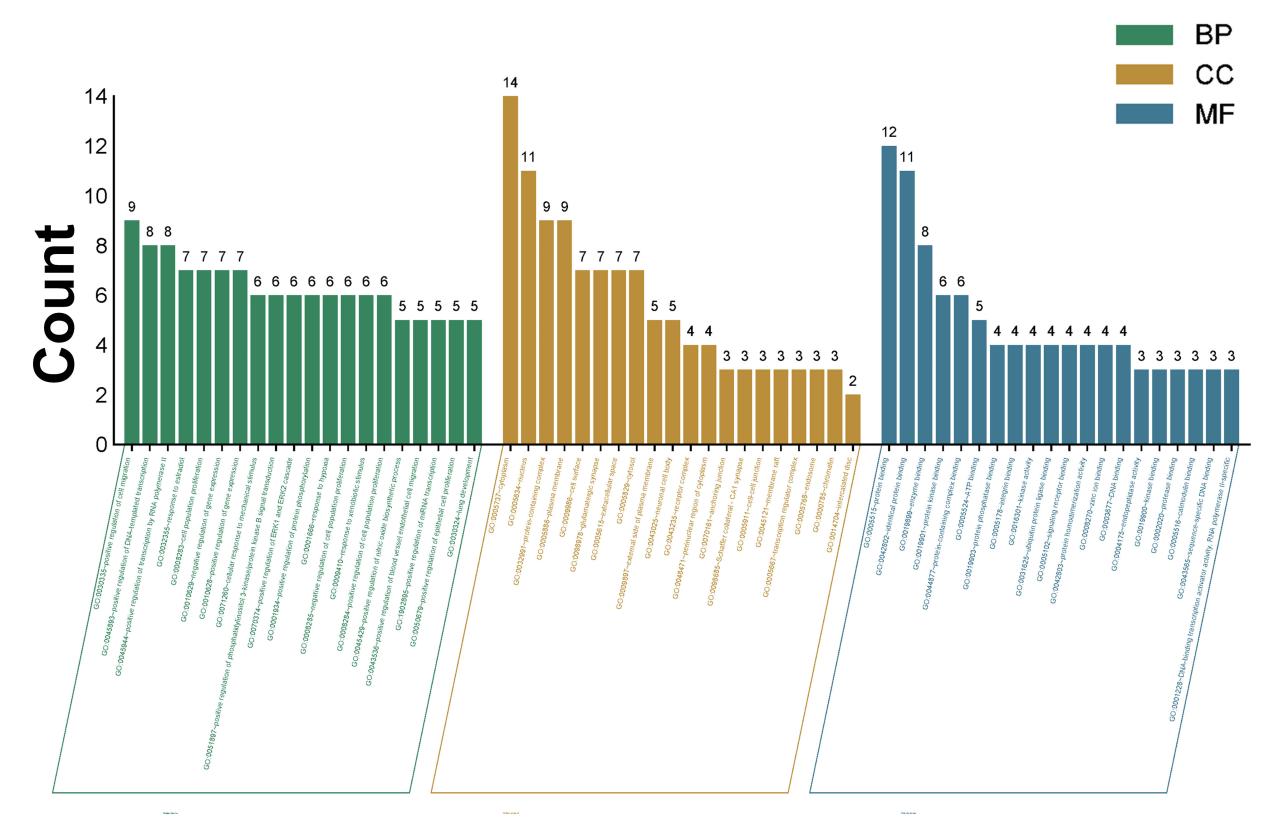


Fig. S6 The GO enrichment analysis of AS-IV@HdECM against MI.


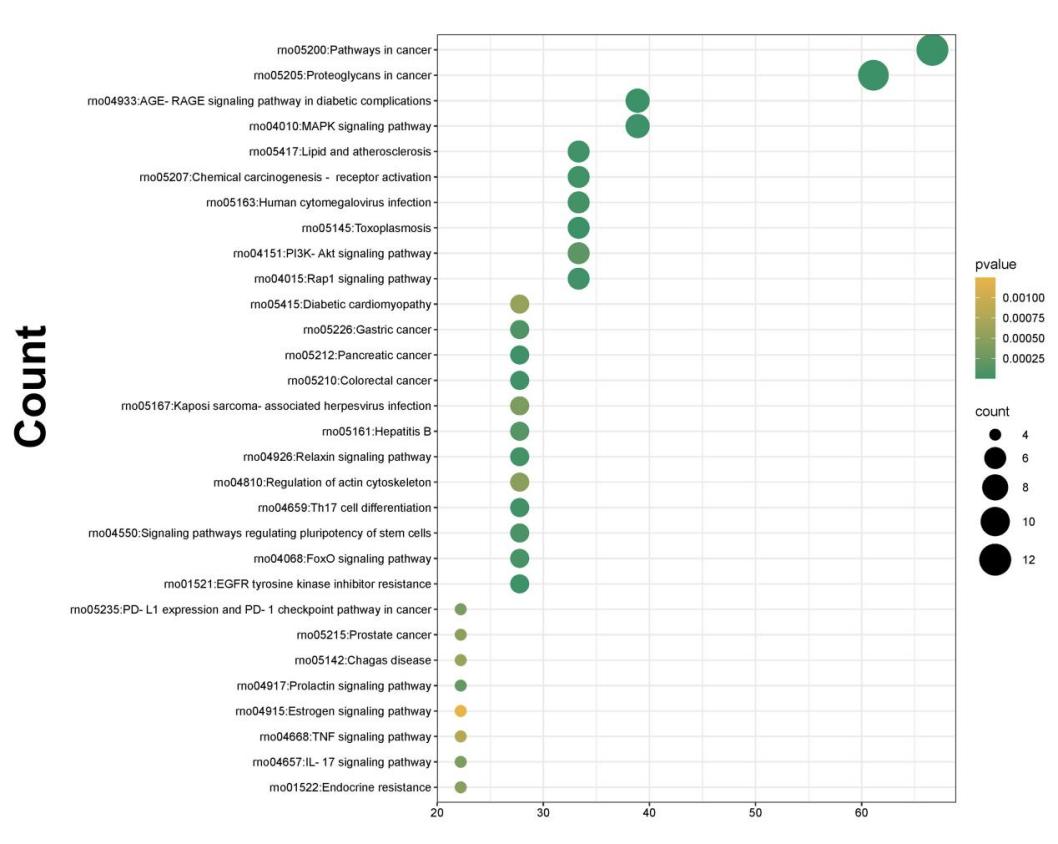


Fig.S7 The KEGG enrichment analysis of AS-IV@HdECM against MI.
